# Robotic and laparoscopic liver resection—comparative experiences at a high-volume German academic center

**DOI:** 10.1007/s00423-021-02152-6

**Published:** 2021-04-08

**Authors:** E. Lorenz, J. Arend, M. Franz, M. Rahimli, A. Perrakis, V. Negrini, A. A. Gumbs, R. S. Croner

**Affiliations:** 1 Department of General, Visceral, Vascular, and Transplant Surgery, University Hospital Magdeburg, Leipziger Strasse 44, 39120 Magdeburg, Germany; 2 Centre Hospitalier Intercommunal de Poissy/Saint-Germain-En-Laye, 10 Rue du Champ Gaillard, 78300 Poissy, France

**Keywords:** Minimally invasive, Liver surgery, Hepatectomy, Robotic, CRC, HCC

## Abstract

**Purpose:**

Minimally invasive liver surgery (MILS) is a feasible and safe procedure for benign and malignant tumors. There has been an ongoing debate on whether conventional laparoscopic liver resection (LLR) or robotic liver resection (RLR) is superior and if one approach should be favored over the other. We started using LLR in 2010, and introduced RLR in 2013. In the present paper, we report on our experiences with these two techniques as early adopters in Germany.

**Methods:**

The data of patients who underwent MILS between 2010 and 2020 were collected prospectively in the Magdeburg Registry for Minimally Invasive Liver Surgery (MD-MILS). A retrospective analysis was performed regarding patient demographics, tumor characteristics, and perioperative parameters.

**Results:**

We identified 155 patients fulfilling the inclusion criteria. Of these, 111 (71.6%) underwent LLR and 44 (29.4%) received RLR. After excluding cystic lesions, 113 cases were used for the analysis of perioperative parameters. Resected specimens were significantly bigger in the RLR vs. the LLR group (405 g vs. 169 g, *p* = 0.002); in addition, the tumor diameter was significantly larger in the RLR vs. the LLR group (5.6 cm vs. 3.7 cm, *p* = 0.001). Hence, the amount of major liver resections (three or more segments) was significantly higher in the RLR vs. the LLR group (39.0% vs. 16.7%, *p* = 0.005). The mean operative time was significantly longer in the RLR vs. the LLR group (331 min vs. 181 min, *p* = 0.0001). The postoperative hospital stay was significantly longer in the RLR vs. the LLR group (13.4 vs. LLR 8.7 days, *p* = 0.03). The R0 resection rate for solid tumors was higher in the RLR vs. the LLR group but without statistical significance (93.8% vs. 87.9%, *p* = 0.48). The postoperative morbidity ≥ Clavien-Dindo grade 3 was 5.6% in the LLR vs. 17.1% in the RLR group (*p* = 0.1). No patient died in the RLR but two patients (2.8%) died in the LLR group, 30 and 90 days after surgery (*p* = 0.53).

**Conclusion:**

Minimally invasive liver surgery is safe and feasible. Robotic and laparoscopic liver surgery shows similar and adequate perioperative oncological results for selected patients. RLR might be advantageous for more advanced and technically challenging procedures.

## Introduction

The first reported laparoscopic wedge resection of the liver was performed in 1992 in Glasgow, UK. Nevertheless, open liver surgery remained the “gold standard” of treatment for quite some time, and Germany especially was a late adopter [1]. In an early expert consensus, laparoscopic liver surgery was recommended for solitary lesions of five or less centimeters and for locations from segment 2 to 6 only [2]. Indeed, the posterior liver segments 7 and 8 are more challenging for minimally invasive liver surgery (MILS) because of the limited reach of laparoscopic instruments. With a better understanding of the technical demands of MILS and improved laparoscopic visualization and multifunctional instruments, MILS has now gained more acceptance among surgeons worldwide. Shorter operative time, reduced postoperative pain, less blood loss, and thus reduced transfusion requirements as well as shorter hospitalizations are known advantages of laparoscopic liver resections (LLR) compared to open liver resections (OLR) [3, 4]. Nevertheless, there has been persistent discussion that due to a relevant learning curve for MILS, these reported advantages might have been the result of a selection bias from easier cases, especially in early studies. However, recent prospective randomized controlled trials have validated the decreased morbidity and hospitalization of patients who underwent MILS compared to open procedures [5].

In 2008, during a consensus conference in Louisville, KY, USA, 45 experts in hepatobiliary surgery confirmed LLR as being feasible, safe, and effective when performed by hepatobiliary surgeons with expertise in laparoscopy. An extensive operative experience is crucial before centers begin performing minimally invasive major liver resections [2]. In 2009, Nguyen et al. published the first comprehensive meta-analysis on LLR. They included 127 articles involving a total of 2804 patients. They reported conversion rates to open surgery of about 4.1%, and a postoperative mortality rate of 0.3% without any intraoperative deaths. R0 resection margins were achieved in 82 to 100% of the cases. When comparing 3- and 5-year survival rates to open liver surgery, they concluded that LLR can be performed with acceptable mortality and similar oncological outcomes [6]. LLR was also used for repeated laparoscopic liver resections in patients with a history of OLR or LLR, but an increased conversion rate of up to 11% was noted. While blood loss was higher and operative time was longer when performing repeated laparoscopic liver resections after initial open liver surgery, hospital stay and perioperative morbidity rates were independent of the initial operative approach [7].

A report on the first robotic liver resection (RLR) was published in 2003 by Giulianotti et al. [8]. RLR adds new technical innovations to conventional laparoscopy which might improve the ability to perform more advanced cases of MILS. In a recent international consensus statement, RLR was considered a safe procedure for minimally invasive liver resections [9]. But there has been an ongoing debate on whether RLR is superior or inferior to LLR in terms of perioperative outcome, cost effectiveness, and/or oncological parameters. Montalti et al. published the first meta-analysis comparing perioperative results among patients undergoing either RLR or LLR [10]. LLR showed significantly shorter operative times and less blood loss. Nevertheless, positive resection margins (R1), perioperative morbidity, conversion rate, and total length of hospital stay were equal between both groups.

Challenges in robotic surgery, including high costs, longer setup times, and mandated structured training programs, might be the reason for the slow adoption of RLR worldwide. We started using robotic liver surgery in 2013 as the first group in Germany after a learning period using LLR [11–13]. As early adopters, we share our experiences with both robotic and laparoscopic liver resections.

## Material and methods

### Patients and selection criteria

MILS has been performed at the Department of General, Visceral, Vascular and Transplant Surgery, University Hospital Magdeburg, since 2010. We initially started performing liver cyst resections, followed by minor resections, and then started doing major liver resections for malignant tumors (primary liver tumors, hepatic metastases). The extent of liver resection in each case was categorized in accordance with “The Brisbane 2000 terminology of Liver Anatomy and Resection” [14]. A major liver resection was defined as a resection of three or more segments including (extended) left or right hemihepatectomies. From 2010 to 2014, we followed mainly the recommendations of Buell et al. with regard to the selection criteria for LLR [2]. Since 2015, we have only excluded cases from MILS which showed major vascular infiltration and which were considered for vascular reconstruction. More technically challenging and advanced cases were transferred to RLR [15, 16]. We established the Magdeburg MILS registry (MD-MILS) collecting all data of patients undergoing laparoscopic (LLR) or robotic (RLR) liver resection.

After obtaining approval from our institutional review board (IRB), we prospectively collected perioperative data of all consecutive patients who underwent LLR or RLR between January 2010 and April 2020. Patients undergoing liver resection following liver trauma, liver biopsy, or laparoscopic ablation were excluded from this analysis. We included data of all patients over 18 years of age undergoing minor or major MILS for malignant or benign lesions. In-hospital morbidity was assessed using the Clavien-Dindo classification and the 30-day mortality rates were analyzed. During pathological examination of the resected specimens, the total number of resected lesions, the maximum measured lesion size, and the resection margins were examined.

### Preoperative patient assessment

All patients received a preoperative clinical assessment by a physician, including laboratory tests to evaluate their liver function. In our department, esophagogastroduodenoscopy and colonoscopy are mandatory prior to liver surgery for solid liver tumors. Hereby, we exclude synchronous secondary malignancies prior to surgery. All patients underwent staging computed tomography (CT) or MRI scans prior to liver resection. In order to minimize the risk of postoperative liver failure, we calculated a computerized future remnant liver volumetry using CT/MRI scans. Additionally, the maximum liver function capacity was tested using the LiMAx test in selected patients [17, 18].

### Minimally invasive liver surgery

LLR was carried out as a totally laparoscopic procedure. No hand-assisted or hybrid techniques were used. The trocars were placed depending on the site of liver resection as described previously [19–21]. For parenchymal dissection, we used a harmonic scalpel and a laparoscopic CUSA (cavitron ultrasonic surgical aspirator) or an aquajet. The specimens were placed in a retrieval bag and removed via a Pfannenstiel incision. For RLR, the Da Vinci System (Intuitive, Santa Clara, USA) was used, i.e., the Si, X, or Xi system during the period of this study. All robotic cases were performed using techniques as described previously [11, 22].

### Difficulty scoring for minimally invasive liver surgery

In order to address the complexity of liver resection, we used the criteria introduced by Wakabayashi et al. in 2016. These form a modified liver resection difficulty score that is based on a score initially introduced by Ban et al. in 2014 [15, 16]. This scoring system uses preoperatively collected data, such as tumor location, tumor size of less than 3 cm or 3 and more cm, presence of Child A/B liver cirrhosis, proximity to major vessels (main or second-order Glissonian pedicles, major hepatic veins, or inferior vena cava), extent of liver resection, and usage of hybrid/hand-assisted surgical techniques.

We defined “proximity to major vessels” as tumors located less than 1 cm away from relevant vascular structures in given preoperative sectional imaging. The complete scoring system was described elsewhere [15, 23, 24]. The total score (0–12 points) was calculated and the following difficulty levels were defined: low difficulty (1–3 points), intermediate difficulty (4–6 points), and high difficulty (requiring advanced/expert surgeons, > 6 points).

### Statistical analysis

Intraoperative and perioperative parameters between both cohorts (laparoscopic liver resection and robotic liver resection) were compared (Tables 1 and 2). Statistical analysis was performed using the SPSS Version 24 software package (IBM Corporation, Armonk, NY, USA). In univariate analysis, categorical variables (nominal/ordinal) are presented as absolute (*n*) and/or relative values (%). Differences between the groups were tested using the Pearson’s *χ*^2^ or Fisher’s exact test (if at least one cell had a cell count of less than 5). Continuous variables were expressed as a mean (SD—standard deviation) or median (IQR—interquartile range), as appropriate. Differences between continuous variables were tested using the Student’s *t*-test or Mann–Whitney *U* test depending on the scale level. For all analyses, differences with a two-sided *p*-value < 0.05 were considered to be significant (no adjustment for multiplicity).

**Table 1:** Demographics and clinical characteristics of patients receiving laparoscopic (LLR) or robotic liver resection (RLR) for a malignant or benign disease; data from the Magdeburg Registry for Minimally Invasive Liver Surgery (MD-MILS)

	LLR *n* = 111	RLR *n* = 44	*p*-value
Age; mean ± SD [years]	61.7 ± 15.3	62.6 ± 14.5	0.75
Gender; *n* (%)			0.4
Female	61 (55.0)	20 (45.5)	
Male	50 (45.0)	24 (54.5)	
BMI; mean ± SD [kg/m^2^]	27.0 ± 4.6	26.5 ± 3.9	0.61
ASA Score; mean ± SD	2.3 ± 0.7	2.3 ± 0.7	0.88
Prior abdominal surgery; *n* (%)	39 (35.5)	28 (63.6)	**0.001**
Liver cirrhosis; *n* (%)	23 (24.5)	9 (20.9)	0.6
Disease; *n* (%)			
Malignant	58 (52.3)	32 (72.7)	**0.02**
Hepatocellular carcinoma	33 (56.9)	13 (40.6)	
Colorectal liver metastases	12 (20.7)	12 (37.5)	
Intrahepatic cholangiocarcinoma	4 (6.9)	5 (15.6)	
Other	9 (15.5)	2 (6.2)	
Benign	53 (47.7)	12 (27.3)	
Cystic lesions	39 (73.6)	3 (25)	
Focal nodular hyperplasia	8 (15.1)	4 (33.3)	
Hemangioma	3 (5.7)	1 (8.3)	
Hepatocellular adenoma	2 (3.8)	2 (16.7)	
Other	1 (1.9)	2 (16.7)	
Pathology report on solid tumors^1^			
Number of lesions; *n* (%)			0.25
1 tumor	52 (72.3)	30 (73.2)	
2 tumors	11 (15.5)	3 (7.3)	
3 or more tumors	8 (11.1)	7 (17)	
No vital tumor in specimen	0	1 (2.4)	
Diameter of largest tumor; mean ± SD (cm)	3.7 ± 2.4	5.6 ± 2.7	**0.001**
Specimen weight; mean ± SD (g)	169.8 ± 198.4	405.1 ± 352.7	**0.002**
R0-margin, if malignancy; *n* (%)	51 (87.9)	30 (93.8)	0.48

*BMI*, body mass index; *ASA*, American Society of Anesthesia; ^1^excluding cystic lesions

**Table 2:** Perioperative parameters of patients receiving laparoscopic (LLR) or robotic liver resection (RLR) for solid tumors; data from the Magdeburg Registry for Minimally Invasive Liver Surgery (MD-MILS)

	LLR *n* = 72	RLR *n* = 41	*p*-value
Extent of liver resection; *n* [%]			
Major resection (≥ 3 segments)	12 (16.7)	16 (39.0)	**0.005**
Minor resection	60 (83.3)	25 (61.0)	
Anatomic tumor location^1^; *n* (%)			**0.002**
Anterolateral	56 (77.8)	21 (51.2)	
Posterosuperior	3 (4.2)	1 (2.4)	
Combined locations	13 (18.1)	19 (46.3)	
Difficulty score^2^; mean ± SD	4.8 ± 2.4	6.5 ± 2.2	**< 0.001**
Difficulty level^3^; *n* (%)			**0.04**
Low difficulty (1–3 score points)	21 (29.2)	5 (12.2)	
Intermediate difficulty (4–6 score points)	31 (43.1)	16 (39)	
High difficulty (> 6 points)	20 (27.8)	20 (48.8)	
Measured blood loss; mean ± SD (ml)	425.4 ± 590.1	439.8 ± 346.3	0.89
Intraoperative blood transfusion; *n* (%)	7 (9.7)	7 (17.5)	0.25
Operative time; mean ± SD (min)	181.3 ± 100.4	330.5 ± 132.2	**0.0001**
Postoperative hospital stay; mean ± SD [d]	8.7 ± 5.8	13.4 ± 12.5	**0.03**
Total postoperative stay ICU; mean ± SD (day)	0.9 ± 1.7	3.1 ± 9.8	0.16
Postoperative morbidity Clavien-Dindo ≥ grade 3; *n* (%)	4 (5.6)	8 (17.1)	0.1
Morbidity; *n* (%)			0.57
Surgical complications	3 (4.2)	4 (9.7)	
Non-surgical complications	1 (1.3)	4 (9.7)	
In-hospital mortality; *n* (%)	2 (2.8)	0	0.53

^1^Anatomic tumor location: Anterolateral, liver segments 2, 3, 4b, 5, 6; posterosuperior, liver segments 4a,7,8; combined locations, parts of anterolateral and posterosuperior segments including hemihepatectomies

^2^Difficulty score according to the “difficulty scoring system for laparoscopic liver resection” introduced by Wakabayashi et al. in 2016

^3^Difficulty level according to calculated difficulty score introduced by Wakabayashi et al.; *ICU*, intensive care unit; surgical complication, bleeding, perforation, abdominal fluid collection, biliary leakage; non-surgical complications, pulmonary embolism, pneumonia, kidney failure

In a case-control approach, all patients who received either LLR or RLR for solid tumors were matched 1:1 by propensity score analysis. Matching criteria were the following: age, gender, sex, prevalence of liver cirrhosis, difficulty score of resection, number of lesions, and type of disease.

## Results

### Patient characteristics

**Fig. 1:**
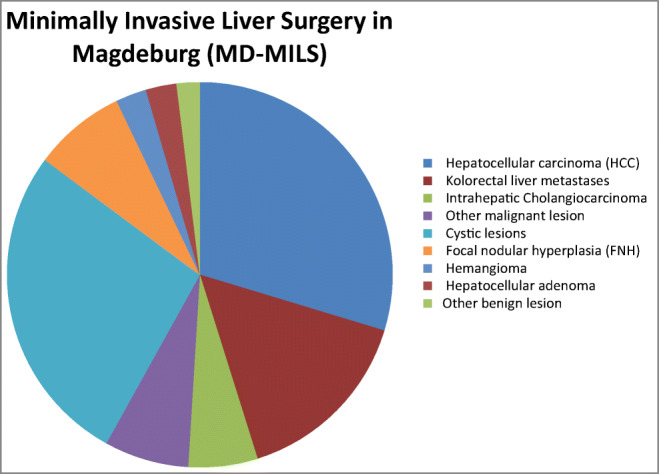
Total malignant and benign liver lesions for laparoscopic (LLR) or robotic liver resection (RLR); data from the Magdeburg Registry for Minimally Invasive Liver Surgery (MD-MILS) between 2010 and 2020

**Fig. 2:**
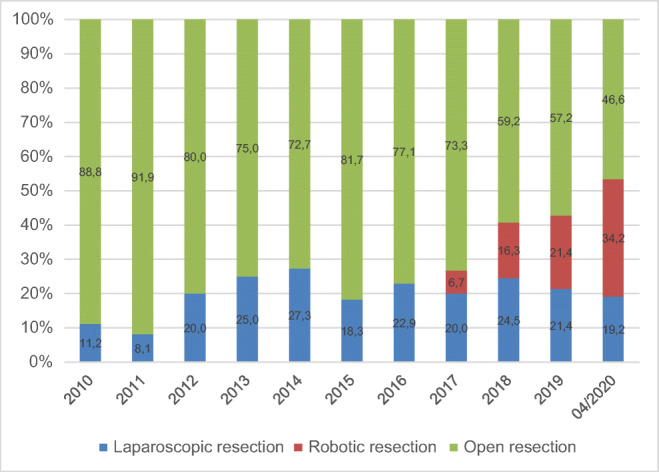
Development of minimally invasive liver surgery at the University Hospital Magdeburg between 2010 and 2020, given values are presented in percent

**Fig. 3:**
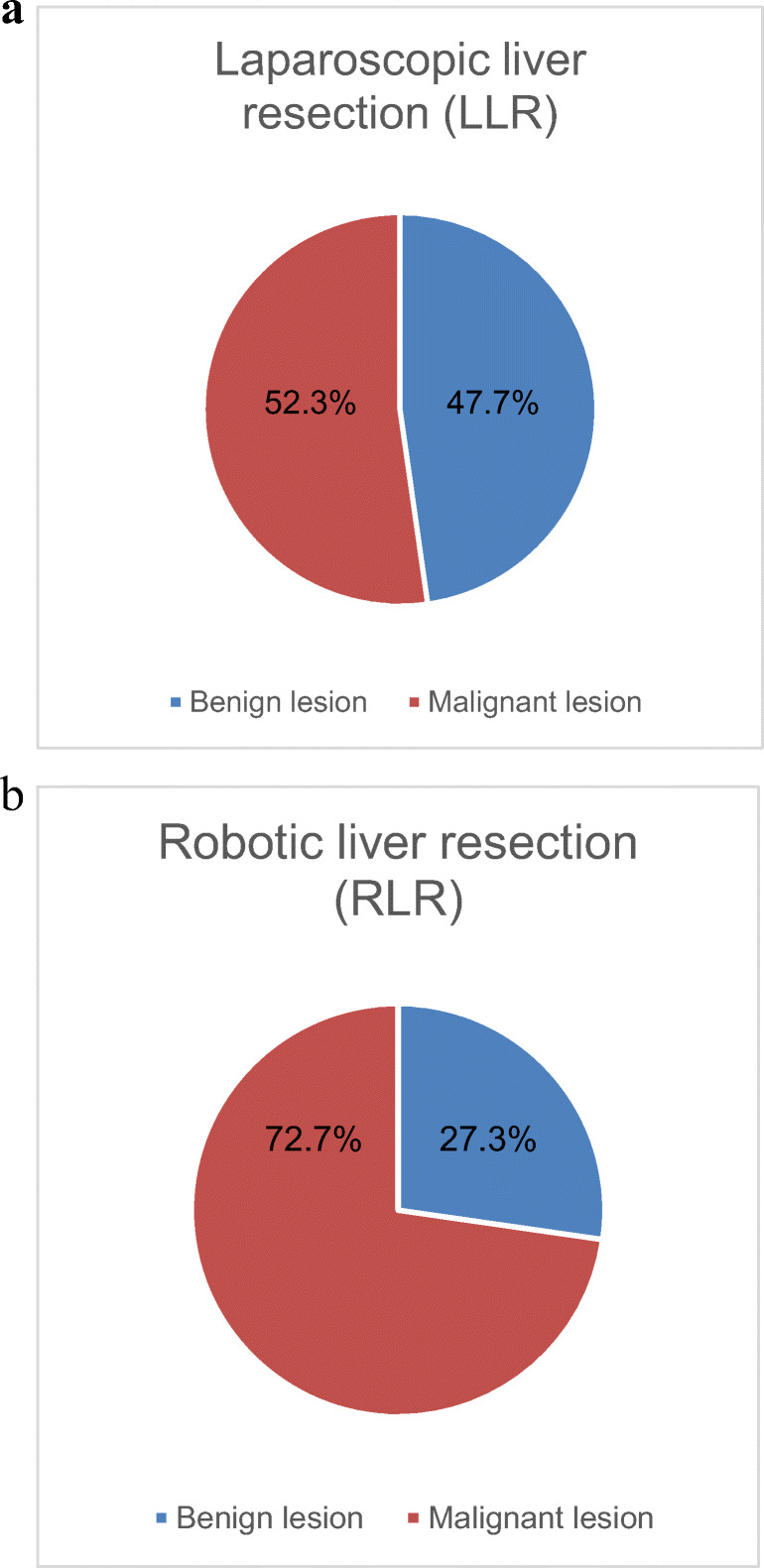
Proportion of malignant and benign liver lesions in laparoscopic (LLR) (**a**) or robotic liver resection (RLR) (**b**); data from the Magdeburg Registry for Minimally Invasive Liver Surgery (MD-MILS), benign lesions include liver cysts

A total number of 155 patients fulfilled the inclusion criteria and underwent MILS procedures from January 2010 to April 2020 (Table 1; Fig. 1). We identified 111 (71.6%) LLR procedures and 44 (29.4%) RLR cases. During the last 3 years, the number of MILS has increased to more than 50% of our total cohort. The rate of RLR has increased from 5 to 22% (Fig. 2). Between the LLR group and the RLR group, no significant differences in mean age or gender distribution were identified. In addition, mean body mass index (BMI) and mean American Society of Anesthesia score (ASA) did not differ significantly. The prevalence of liver cirrhosis was 24.5% in the LLR group and 20.9% in the RLR group, and this difference was not significant. There was no significant difference in the Child–Pugh classification or MELD score between the two groups (data not shown). The prevalence of previous abdominal surgical procedures was 63.6% in the RLR group and thus nearly twice as high as compared to the LLR group (35.5%, *p* = 0.001). RLR was significantly more frequently used for resection of malignant lesions (RLR 72.7% vs. LLR 52.3%, *p* = 0.02; Fig. 3).

### Histopathology and tumor characteristics

The number of resected tumors did not differ between the LLR and the RLR group. Nevertheless, the tissue weight of the resected specimens was significantly higher in the RLR compared to the LLR group (405 g vs. 169 g, *p* = 0.002), and even the maximum diameter of the biggest tumor lesion was significantly larger in the RLR than in the LLR group (5.6 cm vs. 3.7 cm, *p* = 0.001). More primary and secondary malignant tumors were resected in the RLR vs. the LLR group (73% vs. 52%; *p* = 0.02). The R0 resection rate of malignant tumors was higher in the RLR vs. the LLR group, but this did not have any statistical significance (93.8% vs. 87.9%, *p* = 0.48) (Table 1).

### Perioperative outcomes of MILS in solid tumors

We excluded cystic tumors from the analysis of perioperative outcomes because these patients represented the least difficult cases and were mainly operated during the initial learning curve. Thus, 113 patients with solid tumors were included into this analysis (Table 2). The number of major resections was significantly higher in the RLR vs. the LLR group (39.0% vs. 16.7%, *p* = 0.005). Consequently, anatomic tumor location differed significantly between both groups. In the LLR group, 77.8% of all resections strictly involved the anterolateral segments (liver segments 2, 3, 4b, 5, 6), while in the RLR group almost half (46.3%) of all resections were combined resections involving the upper segments (liver segments 4a, 7, 8). This difference was significant between the RLR vs. the LLR group (*p* = 0.002). The mean operative time was significantly longer in the RLR vs. the LLR group (331 min vs. 181 min, *p* = 0.0001). The postoperative hospital stay was significantly longer in the RLR vs. the LLR group (RLR 13.4 vs. LLR 8.7 days, *p* = 0.03). We identified a significantly longer hospitalization in major liver resection vs. minor liver resection when both laparoscopic and robotic cases were combined (major resection 14.5 days vs. minor resection 9.0 days, *p* = 0.04). The incidence of Clavien-Dindo grade 3a to 5 complications did not differ significantly between the LLR and the RLR group (*p* = 0.1).

In the LLR group, four severe postoperative complications were identified. Three of them were classified as surgical complications including two patients with a postoperative intra-abdominal fluid collection requiring CT-guided percutaneous drainage. One patient suffered a duodenal perforation caused by a postoperative ulcer and needed reoperation, but the patient died due to postoperative peritonitis with sepsis. The remaining non-surgical complication was diagnosed as a pulmonary embolism, and despite cardiopulmonary resuscitation the patient expired.

The RLR group had eight postoperative complications > Clavien-Dindo grade 2. Four of those complications were of surgical origin. There were two Clavien-Dindo grade 3a complications: one biliary leakage from the resected area requiring endoscopic retrograde cholangiopancreatography (ERCP) and one fluid collection necessitating CT-guided percutaneous drainage. There were two instances of Clavien-Dindo grade 3b complications in the RLR group: one postoperative bleeding from the inferior vena cava and one gastric perforation. Both cases needed reoperation. Four cases of non-surgical complications were identified: two pulmonary embolisms, one pneumonia, and one acute-on-chronic kidney failure requiring hemodialysis. All of these four cases required temporary ICU care.

No patient in the RLR group (0%) but two patients in the LLR group (2.8%) died during their hospitalization for MILS (Table 2).

In a propensity score analysis, we matched 40 patients of the LLR and 40 patients of the RLR group. Here, we identified no significant differences in postoperative morbidity (Clavien-Dindo ≥ grade 3), in-hospital mortality or in intensive care unit stay between the groups.

### Major laparoscopic and major robotic liver resection

Comparing LLR and RLR for major liver resection (resection of three or more segments), there were no significant differences in patient characteristics such as gender, age, BMI, or ASA score (Table 3). Patients receiving major liver resection via RLR significantly more often had a history of previous abdominal surgery compared to the LRL patients (87.5% vs. 41.7%; *p* = 0.02). Mean operation time was significantly longer in the RLR group (RLR = 409 min vs. LLR = 270 min; *p* = 0.01). However, it must be pointed out that the difficulty score was significantly higher in the RLR group (RLR = 8.6 vs. LLR = 7.3; *p* = 0.04). Tumor size (6.6 cm vs. 5.0 cm) and volume of resected liver tissue (656 g vs. 424 g) were also higher in the RLR group but without statistical significance. Referring to blood loss (RLR = 503 ml vs. LLR = 941 ml; *p* = 0.2) in major liver resection, RLR might be favorable. The length of hospital stay was significantly longer in the RLR group (19.0 days vs. 8.5 days; *p* = 0.02). Morbidity or in-hospital mortality did not differ significantly between these groups.

**Table 3:** Demographics and clinical characteristics of patients receiving major liver resection (≥ 3 segments) by means of laparoscopic (LLR) or robotic liver resection (RLR) for solid tumors; data from the Magdeburg Registry for Minimally Invasive Liver Surgery (MD-MILS)

	LLR *n* = 12	RLR *n* = 16	*p*-value
Age; mean ± SD (years)	68.2 ± 9.6	67.1 ± 13.1	0.8
Gender; *n* (%)			0.1
Female	2 (16.7)	8 (50)	
Male	10 (83.3)	8 (50)	
BMI; mean ±SD (kg/m^2^)	26.1 ± 2.8	25.7 ± 4.1	0.8
ASA Score; mean ± SD	2.4 ± 0.5	2.4 ± 0.7	0.9
Prior abdominal surgery; *n* (%)	5 (41.7)	14 (87.5)	**0.02**
Liver cirrhosis; *n* (%)	3 (25)	2 (12.5)	0.62
Disease; *n* (%)			
Malignant	12 (100)	15 (93.7)	
Hepatocellular carcinoma	5 (41.7)	6 (37.5)	
Colorectal liver metastases	4 (33.3)	5 (31.3)	
Intrahepatic cholangiocarcinoma	1 (8.3)	3 (18.8)	
Other metastases	2 (16.7)	1 (6.3)	
Benign	0 (0)	1 (6.3)	
Focal nodular hyperplasia	0 (0)	1 (6.3)	
Diameter of largest tumor; mean ± SD (cm)	5.0 ± 2.5	6.6 ± 2.4	0.1
Specimen weight; mean ± SD (g)	424.0 ± 258.0	656.0 ± 374.8	0.1
R0-margin, if malignancy; *n* (%)	10 (83.3)	14 (93.3)	0.6
Measured blood loss; mean ± SD (ml)	941.7 ± 1116.6	503.1 ± 387.1	0.2
Operative time; mean ± SD (min)	269.6 ± 130.1	408.7 ± 128.2	**0.01**
Iwate difficulty score^1^; mean ± SD	7.3 ± 2.0	8.6 ± 1.2	**0.04**
Postoperative hospital stay; mean ± SD (day)	8.5 ± 3.7	19.0 ± 15.4	**0.02**
Total postoperative stay ICU; mean ± SD (day)	1.8 ± 1.8	6.8 ± 15.05	0.3
Morbidity Clavien-Dindo ≥ grade 3; *n* (%)	1 (8.3)	4 (25)	0.4
In-hospital mortality; *n* (%)	1 (8.3)	0 (0)	0.4

^1^Difficulty Score according to the “Difficulty Scoring system for laparoscopic liver resection” introduced by Wakabayashi et al. in 2014; *ICU*, intensive care unit

## Discussion

Our findings confirm the safety and efficacy of minimally invasive liver surgery including LLR and RLR for minor and major liver resections in the treatment of malignant and benign solid hepatic tumors. A recent meta-analysis concluded that LLR and RLR are both equally feasible and effective regarding the oncological outcomes [25]. In 2018, an international expert group of hepatobiliary surgeons published a consensus statement declaring the equivalency of both minimally invasive techniques for liver resection; however, a lack of randomized controlled trials was noted [9]. Furthermore, another paper advised to overcome the learning curve of RLR before embarking on using robotic major liver resection for cases of malignant disease [26] in order to ensure patients’ safety, a reduced operative time, a reduced blood loss, and a high oncological quality. Two studies showed that after 25–30 robotic liver resections, the achieved perioperative outcomes might be superior to LLR [27, 28]. However, it has to be noted that these results might depend on the surgeon’s previous experience in laparoscopic liver surgery.

In elective liver surgery, we demonstrated that with robotic support, it is feasible to resect larger tumors including more liver tissue and to achieve comparable or potentially even better R0 margins compared to LLR. The reason for this finding is the selection bias in our robotic group where we performed proportionally more major liver resections and more resections of the deep segments. These procedures have been classified as being more advanced cases for MILS [16]. Sometimes, it is more challenging to remove single small lesions deep inside the liver than big lesions, which are located in more accessible areas of the liver. We addressed this issue by including a difficulty scoring in our analysis, which identified significantly more difficult cases in the RLR group. Nevertheless, we found no significant differences in blood loss, morbidity, and mortality between the RLR and the LLR group. This is a clear indicator that the robot is a tool, which enables the surgeon to handle even complex liver resections. Our data is in concordance with the results of a meta-analysis on RLR vs. LLR including 776 patients. Qui et al. reported an overall morbidity rate of 11.4% without significant differences between the RLR and the LLR group, whereas major liver resections were performed more frequently in the RLR group (54.7%) compared to the LLR group (25.2%) [29]. The increased postoperative hospitalization and the longer operative times in the RLR vs. the LLR group are explained by the fact that more advanced cases were selected for the robotic procedure in our study. In a subgroup analysis, we confirmed that in the major resection group, operative time was extended and patients needed a longer time to recover from surgery when compared to minor liver resections. Notably, these results apply for LLR in addition to RLR [30]. Nevertheless, we identified a slightly higher amount of blood loss in the RLR group as described in previous meta-analyses comparing robotic to laparoscopic liver resection [10, 25]. Importantly, in our cohort, the blood loss was measured and not estimated as in other reports.

There has been an ongoing discussion on whether a history of previous abdominal surgery inducing adhesions might influence the perioperative outcome of MILS. Van der Poel et al. reported that in patients with previous liver surgery, MILS is as safe for resections of liver metastases as are open procedures [31]. Park et al. reported that adhesions do not influence perioperative morbidity in cases of laparoscopic or robotic resections for colorectal cancer [32]. In a study regarding robotic cystectomy, previous abdominal surgery influenced postoperative morbidity significantly [33]. The safety of robotic surgery, especially in cases of solid liver tumors in patients with abdominal adhesions, has not been proven extensively so far. We did not find any significant difference in perioperative morbidity and mortality between the groups in our cohort. It should be noted that patients in the RLR group suffered from abdominal adhesions almost twice as often compared to patients in the LLR group (63.6% vs. 35.5%). We concluded that previously performed open abdominal surgery is not a contraindication for MILS and especially not for RLR.

Currently, the standard of surgery for liver tumors in Germany is still open liver surgery. Comparing our perioperative data of MILS with recently published results of 110,332 cases of patients who underwent liver surgery in Germany between 2010 and 2015, several differences were identified [34]. MILS for minor hepatectomy in our cohort led to a perioperative mortality of 1.2% compared to the in-hospital mortality of up to 3.8% described by Filmann et al. [34]. In our patient population, perioperative mortality following MILS for major hepatectomy was 3.6% and thus still below the average mortality of 10.4% mentioned by Filmann et al. [34]. Risk factors such as age > 50 years and male gender resulting in an elevated level of perioperative mortality (4.3–6.9%) were described by Filmann et al. [34]. In our major resection group, the mean age was 67.6 years and included 64.3% male patients. Thus, in our study, we selected a higher risk group of patients for a more advanced procedure (MILS) performed more often using the robot but with reduced mortality compared to the findings of Filmann et al. (3.6% vs. 10.4%), respectively. These facts indicate that a paradigm shift towards minimally invasive approaches for liver resection should be considered in Germany. It has to be mentioned that the 90-day mortality of our total cohort (including open cases) was 4.9%.

Nevertheless, our study has several limitations. LLR was performed by several surgeons with different levels of experience, while robotic liver resection (RLR) was performed by a single surgeon already experienced in LLR prior to beginning RLR. Therefore, the results include learning curves which may bias the peri- and postoperative outcomes. We are aware that the retrospective non-randomized study design might contribute to a selection bias. We also acknowledge that the relatively low statistical power of our study might lead to a misinterpretation of results and that the statistical power needs to be increased in the future. Furthermore, the German healthcare system requires a predefined length of hospitalization for reimbursement. This influences the postoperative stay which is elevated in our cohort when compared to many other international centers [10, 25].

## Conclusion

In summary, we confirmed the safety and feasibility of MILS for benign and malignant liver tumors. The robot was identified as an appropriate tool to ensure high-quality procedures with good perioperative results, especially for major and more complex liver resections. This technique is currently limited by its high costs, a lack of structured fellowships and reimbursement as well as the absence of prospective randomized data. However, our results elucidate that a paradigm shift towards MILS in Germany is indispensable [34].
